# Guiding plant conservation using physiological tools: how mechanistic research can bridge disciplinary divides

**DOI:** 10.1093/conphys/coae090

**Published:** 2025-01-09

**Authors:** Sean Tomlinson

**Affiliations:** Department of Biodiversity, Conservation and Attractions, Biodiversity and Conservation Science, Kensington, WA 6151, Australia; School of Biological Sciences, University of Adelaide, Adelaide, SA 5000, Australia

**Keywords:** Biodiversity conservation, co-design, cross-disciplinary research

## Introduction

Conservation science in the 21st century has become heavily dissected into different fields and subfields, sometimes defined by finely nuanced distinctions, such as the ongoing debate regarding restoration, rewilding, rehabilitation and reafforestation (Aronson *et al.,* 1993; Mutillod *et al.,* 2024). Emergent, and even imaginary, distinctions notwithstanding, all these fields remain unified by principles well defined by Soulé (1985) that ‘Conservation biology, a new stage in the application of science to conservation problems, addresses the biology of species, communities and ecosystems that are perturbed, either directly or indirectly, by human activities or other agents. Its goal is to provide principles and tools for preserving biological diversity’. Similarly, the IUCN (1980), aimed for ‘the management of human use of the biosphere so that it may yield the greatest sustainable benefit to present generations whilst maintaining its potential to meet the needs and aspirations of future generations’. The IUCN (1980) stated that this included the sustainable management of ‘plants, animals and micro-organisms, and those non-living elements of the environment on which they depend’. These definitions of conservation biology have substantial overlap with the founding concepts of wildlife management, that ‘the biotic associations within each park be maintained, or where necessary recreated, as nearly as possible in the condition that prevailed when the area was first visited by the white man.’ (Leopold *et al.,* 1963). Many of the definitions of conservation and ecological management include elements of ecological restoration, which, under various definitions, aims to rapidly return natural ecological structures and processes to landscapes following disturbance (Jackson *et al.,* 1995; Cairns Jr and Heckman, 1996; Miller *et al.,* 2017).

There is a shared philosophical heritage that underpins all the increasingly diverse and finely nuanced sub-disciplines of conservation biology, yet often these disciplines have pursued parallel agendas, with biases towards different taxonomic groups. For example, whilst restoration ecology is often preoccupied with the return of vegetation and plant biodiversity at the expense of animals (Majer, 2009; Cross *et al.,* 2019), Leopold *et al.* (1963) noted that the restoration of ecosystems to promote and support animal populations is essential, but complex. Whilst Soulé (1985) and Caughley (1994) explored the difficulty of biodiversity conservation in managing and recovering small populations of animals, Fiedler and Ahouse (1992) were identifying different syndromes of rarity and exploring the drivers and consequences of small populations in plant conservation. Reintroduction biology has been discussed in relation to animals (Armstrong and Seddon, 2008) and plants (Maschinski and Albrecht, 2017) in parallel, deriving nearly identical guiding principles, but with almost no reference to each other.

Physiology, however, should provide a basic, mechanistic toolset that transcends taxonomic boundaries and informs all aspects of the conservation agenda. Hence, Cooke *et al.* (2013) reasonably defined conservation physiology as a very broad discipline ‘applying physiological concepts, tools and knowledge to characterizing biological diversity and its ecological implications and solving conservation problems across the broad range of taxa (i.e. including microbes, plants and animals)’. Similar recognition of the broad value of physiological data has recently been strongly advocated in restoration ecology (Valliere *et al.,* 2021; Tomlinson *et al.,* 2022). Despite this, there are still taxonomic biases in the literature, and physiological studies tend to align to specific agendas based on taxa. Despite the long heritage of plant physiology as a discipline, plants have generally garnered less attention in the conservation physiology literature (Madliger *et al.,* 2018). Instead, plant physiology research is often couched in an agricultural context, sometimes with a debatable conservation agenda (Giller *et al.,* 2015), or a restoration context (Valliere et al., 2021), with a subset of *ex situ* seed storage, prominently focused on a restoration agenda (Merritt and Dixon, 2011). A lot of animal physiology has a strong bias towards endocrinological studies of stress and reproductive technology in agriculture and threatened species recovery. Even this journal, *Conservation Physiology*, has historically attracted more submissions reporting on animal physiology than any other group (Madliger *et al.,* 2018), although some of this may be the result of a recognized bias in conservation funding (Czech *et al.,* 1998; Clark and May, 2002).

The ‘Traits and Measurements in Plant Conservation’ special issue was developed to increase recognition across taxonomic boundaries of the role that physiological data can play in guiding biodiversity conservation. In inviting submissions, I set a very broad concept of what qualifies as ‘conservation’ in specific recognition of the shared agenda across many related fields. In this way, I specifically sought to bring the attention of plant physiologists to *Conservation Physiology*, but also to encourage a broader shared perspective gained by engaging with the physiological research undertaken across taxonomic divides.

## Major Themes

The link between physiological traits and ecological and evolutionary patterns is an overarching concept underpinning all the manuscripts in the special issue. This concept was approached across three major themes here that are largely consistent with the broader conservation physiology literature: traits and stress resilience, traits and biogeography and *ex situ* conservation.

### Stress resilience

Schönbeck *et al.* (2023) provide a powerful exploration of the value of physiological traits in guiding plant conservation, potentially above and beyond the more typically used phenological or morphological traits. Specifically, the study provides an approach to identify physiological functional impairment as conditions approach the limits of a species’ niche. This study provides insightful evidence that the more mechanistic traits appear in functional traits databases, the stronger the understanding of the interactions between the plant and its environment, and that these interactions drive patterns in ecology, adaptation and biogeography. Valliere *et al.* (2023) provide a great realized example of the principles espoused by Schönbeck *et al.* (2023), combining traditional morphophysical functional traits with an ecophysiological response in the form of leaf fluorescence. This combination provides extra insight into how species might respond to increasing global temperatures, particularly in the case of anthropogenic landscapes characteristic of ecological restoration. Ryan *et al.* (2023) explore the value of incorporating physiological performance traits with microclimatic drivers of germination and emergence in the context of changing fire regimes, finding potential emerging bottlenecks likely to alter population and community structures. Similarly, Harris *et al.* (2024) demonstrate the nuanced insights available by studying physiological traits rather than strictly morphophysical traits when considering adaptability, consistent with a large body of literature exploring similar counterintuitive patterns of acclimation and acclimatization in animals.

### Physiological biogeography

If the first theme that emerged in this special issue was ‘which species will persist and why?’, the second theme that emerged is probably best summarized as ‘where will species persist and why?’ These papers take many of the mechanistic traits advocated by Schönbeck *et al.* (2023) and use them to challenge assumptions around why species are where they are. Rajapakshe *et al.* (2024) capitalize on the long evolutionary history and climatic stability that is uniquely available in many Australian study systems, in comparing the traits that emerge in isolated short-range endemic species. This paper is intriguing because it’s a rare case of turning evolution upside down: although the study species are distinct, and have been distinct for long periods of time, there has been almost no selection on their physiology. This is counter to expectations that physiological changes should underpin the adaptation of species to specific niches. Supplementing a morphophysical understanding of seed dormancy with physiological data, Just *et al.* (2023) demonstrate that alleviation of the physical constraints to germination is difficult to achieve in natural conditions, and likely to occur infrequently under changing climates. The study demonstrates the often conflicting evolutionary drivers that emerge as species are forced into novel niche space. Tudor *et al.* (2024) provide a complex mathematical approach that strongly links functional traits to the biogeographical patterns within a species, clearly demonstrating the mechanistic link between physiological traits and climate. The most comprehensive of these links, however, was provided by Lewandrowski *et al.* (2024), in developing multivariate statistical distribution models of a short-range endemic species to understand the edaphic and microclimatic drivers of habitat suitability, and then using physiological data to validate meaningfulness of the environmental correlates of occurrence.

### *Ex situ* conservation

The final theme that was explored in this special issue is that, for many species, the evidence suggests that they either will not have available habitat in the Anthropocene, or they will not be able to migrate there on their own, as Just *et al.* (2023) data suggest. Some form of conservation in captivity will be required. Gu *et al.* (2024) present a rare study that offers time series insights into seed storage. This provides real data that shows a stark loss of viability (~50%) even in refrigerated *Pinus densiflora* seeds, whilst cryopreserved seeds lost 3% viability. Turner *et al.* (2023) provide a similar exploration of the seed storage paradigm, demonstrating the poor suitability of most storage conditions for a species that is widely collected for *ex situ* conservation and restoration activity. Notably, *Macrozamia fraseri* lost ~50% of their viability in ~5 years (Turner *et al.* (2023)), compared to the *Pinus* species researched by Gu *et al.* (2024), which had equivalent viability loss after 20 years of suboptimal storage. Therefore, species-specific data are still highly valuable, because general synthesized rules are yet to emerge.

## Beyond Botany

The fundamental nature of physiological traits is that they act at the interface between the organism and its environment (Dunham *et al.,* 1989; Bradshaw, 2003). However, physiological traits also allow exploration of biotic interactions and differing responses to similar stimuli across trophic boundaries. In this special issue Wong *et al.* (2024) interrogated such trophic interactions by asking a simple question of whether ‘probiotic’ inoculation with commercially marketed blends of microbial soil enrichment actually improved plant performance in ecological restoration. While the question was simple, the study itself identified a complex series of untested assumptions behind the simple premise of improving restoration outcomes by manipulating biotic interactions. Studies of the physiological consequences of trophic interactions are increasing (Tarszisz *et al.,* 2018; Jones and Rader, 2022) and often provide insights into such unexpected complexities, but overall remain rare in the literature.

A key focus of the research published here is reporting data and identifying trends that can actively advance a conservation agenda. In many cases this has entailed ‘co-design’ or ‘co-production’ of the research, partnering with representatives from industry, community and indigenous groups. Physiological data are gaining traction outside traditional plant physiology disciplines because they provide solid, empirical data that can consequently quantify management thresholds. However, such data take time to accumulate, and often the research that has been published here is the consequence of long-running collaborations.

## Conclusion

Researching physiology provides fundamental mechanistic insights into the interaction between organisms and their environment (Dunham *et al.,* 1989; Bradshaw, 2003). Since physiological traits operate at the interface between evolution and the physicochemical laws that constrain biology, their measurement and analysis provide empirical and mechanistic insight into threatening processes and species’ adaptability, both contemporary and under past and future conditions. Furthermore, however diverse the effector selected by evolution for specific species, these fundamental mechanisms should be highly transferable and broadly informative, because they are responses to nearly universal constraints. These mechanistic insights are fundamental to successful conservation action (Tarszisz *et al.,* 2014; Evans *et al.,* 2015).

*Conservation Physiology* has traditionally received articles biased towards animal models, notwithstanding the broad taxonomic interest identified in the scope of the field (Cooke *et al.,* 2013). Yet measurement tools and analytical interrogation should be applicable across taxonomic divides (Tomlinson *et al.,* 2018b). There are many examples of tools and analyses that bridge these divides well. Respirometry, for example, is widely recognized by most conservation physiologists for its value in animal studies (Frappell, 2006). The same gas analysers are equally effective in measuring plant photosynthesis (Douthe *et al.,* 2018) and can also be used to infer viability and vigour of seeds (Dalziell and Tomlinson, 2017; Tomlinson *et al.,* 2018a). Isotopic analyses, both of stable isotopes of oxygen and hydrogen (Nagy and Costa, 1980; Speakman, 1997) and radioisotopes (Peters *et al.,* 1995; Tomlinson *et al.,* 2013), have substantial heritage in measuring the energetics of free-ranging animals in their natural environments. Yet isotopic analyses are yet to gain substantial traction in plant physiology (Snyder *et al.,* 2022) despite capturing a broader range of physiological processes in plants than animals (Snyder *et al.,* 2022), including trophic interactions between plants and their obligate symbiotes (Davis *et al.,* 2022). Similarly, many animal physiologists would recognize the values of broadly applicable thermal performance curves (Angilletta, 2006), but very few have recognized the early development of a simple and effective thermal performance function to understand the thermal performance of plant photosynthesis (Yan and Hunt, 1999), nor its broad applicability (Tomlinson, 2019) or flexibility to incorporate other environmental pressures (Rajapakshe *et al.,* 2022).

In this special issue, what is hopefully demonstrated is the diversity of conservation physiology as an agenda, with a strong sense of the practical value of the discipline across taxonomic boundaries. While the specific focus of this special issue is plant conservation, the research very much fits the agenda of *Conservation Physiology* and mirrors the themes that have been explored in the context of animal conservation in the past. The three themes here do not capture the full breadth of physiological avenues in research and guiding plant conservation, but they make a solid foundation for a broad body of knowledge to grow from in *Conservation Physiology*.

Madliger *et al.* (2018) noted a relatively limited scope in physiology tools as related to plant conservation and strongly advocated for the development of a comprehensive framework to address this. When I consulted the corresponding authors of each of the contributions to this special issue, they returned with several burning issues and knowledge gaps that they felt need to be addressed:

- The ‘science/policy/practise’ gap remains a problem and can be hugely disruptive where inconsistent funding or collaborations can interrupt the provision of complex physiological data to on-ground practitioners;
- Proxy measures of physiological performance (e.g. those collected by remote sensing) need to be mechanistically linked to processes underpinning population and community structure. Currently, even where physiological traits can be insightful to plant performance, they still are often unable to predict restoration or species recovery outcomes;
- Physiological traits underpinning seed longevity, dormancy and germination triggers are poorly understood, especially for conservation-significant taxa. Currently seed storage is a common tool for conservation and ecological restoration, but we have incomplete data on how best to manage many of these resources to ensure long-term viability;
- Physiological aspects of trophic interactions, such as the passage of seeds through animal guts or nutrient transfer between mycorrhizal fungi and their hosts, on the ecology and evolution of plants need greater attention, especially given their complex, and unpredictable consequences; and
- Physiological traits currently capture snapshots of plant performance under current conditions, or at best recent historical conditions. We lack substantial insights into physiological responses to changing conditions representative of novel niche space, either in anthropogenic landscapes or future anthropogenic climates.

Some of these priorities echo earlier calls (Madliger *et al.,* 2018), demonstrating the persistent challenge in tackling complex questions in conservation biology. Nevertheless, these questions set a broad agenda for the direction of plant conservation physiology for the rest of the 21st century and address some of the challenges inherent in the Anthropocene. They also demonstrate the broad appeal of physiological tools and analyses in applying science to conservation problems. Being situated at the interface between the organism and the environment, physiological data can provide uniquely valuable guidance to managing both the biota and the non-living elements of the environment on which they depend. Hopefully, with this agenda, a greater number of plant physiologists will be adding to the body of conservation physiology literature that we have brought together in this special issue.

## Data Availability

No data sources are associated with this publication.
